# Bis{4-chloro-2-[2-(1*H*-indol-3-yl)ethyl­imino­meth­yl]phenolato-κ^2^ *N*,*O*}zinc(II)

**DOI:** 10.1107/S1600536808002213

**Published:** 2008-01-25

**Authors:** Hapipah M. Ali, M. I Mohamed Mustafa, Mohd. Razali Rizal, Seik Weng Ng

**Affiliations:** a Department of Chemistry, University of Malaya, 50603 Kuala Lumpur, Malaysia

## Abstract

The Zn atom in the title compound, [Zn(C_17_H_14_ClN_2_O)_2_], is *N*,*O*-chelated by two deprotonated Schiff base monoanionic ligands in a tetra­hedral coordination geometry. The Zn atom lies on a special position of site symmetry 2.

## Related literature

For the structure of the unsubstituted [(C_17_H_15_N_2_O)_2_Zn], see Chen *et al.* (2007 ▶); Ng (2008 ▶).
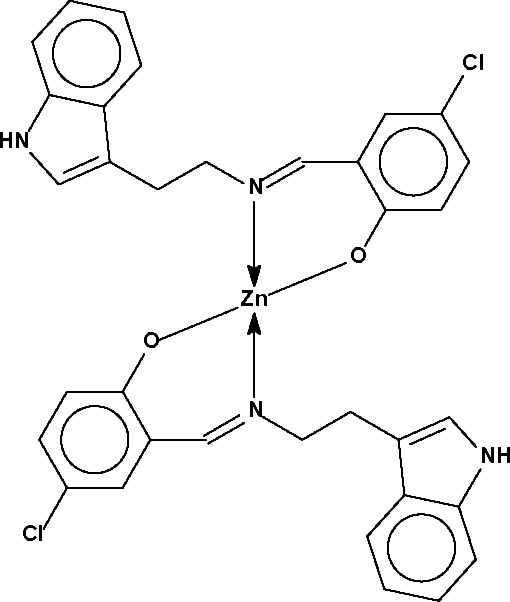


## Experimental

### 

#### Crystal data

[Zn(C_17_H_14_ClN_2_O)_2_]
*M* *_r_* = 660.87
Monoclinic, 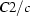

*a* = 25.8989 (3) Å
*b* = 5.4960 (1) Å
*c* = 20.6138 (3) Å
β = 91.801 (1)°
*V* = 2932.73 (8) Å^3^
*Z* = 4
Mo *K*α radiation
μ = 1.06 mm^−1^
*T* = 128 (2) K
0.50 × 0.30 × 0.17 mm

#### Data collection

Bruker APEXII diffractometer
Absorption correction: multi-scan (*SADABS*; Sheldrick, 1996 ▶) *T*_min_ = 0.714, *T*_max_ = 0.840
17664 measured reflections
3352 independent reflections
3023 reflections with *I* > 2σ(*I*)
*R*_int_ = 0.025

#### Refinement

*R*[*F* ^2^ > 2σ(*F* ^2^)] = 0.025
*wR*(*F* ^2^) = 0.100
*S* = 1.21
3352 reflections
251 parameters
14 restraints
All H-atom parameters refined
Δρ_max_ = 0.55 e Å^−3^
Δρ_min_ = −0.56 e Å^−3^

### 

Data collection: *APEX2* (Bruker, 2005 ▶); cell refinement: *SAINT* (Bruker, 2005 ▶); data reduction: *SAINT*; program(s) used to solve structure: *SHELXS97* (Sheldrick, 2008 ▶); program(s) used to refine structure: *SHELXL97* (Sheldrick, 2008 ▶); molecular graphics: *X-SEED* (Barbour, 2001 ▶); software used to prepare material for publication: *publCIF* (Westrip, 2008 ▶).

## Supplementary Material

Crystal structure: contains datablocks global, I. DOI: 10.1107/S1600536808002213/hg2370sup1.cif

Structure factors: contains datablocks I. DOI: 10.1107/S1600536808002213/hg2370Isup2.hkl

Additional supplementary materials:  crystallographic information; 3D view; checkCIF report

## Figures and Tables

**Table:** 

Zn1—O1	1.907 (1)
Zn1—N1	2.016 (1)

**Table:** 

O1—Zn1—O1^i^	116.62 (8)
O1—Zn1—N1	95.57 (5)
O1—Zn1—N1^i^	125.55 (6)
N1—Zn1—N1^i^	99.56 (8)

Symmetry code: (i) 
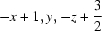
.

**Fig. 1.**
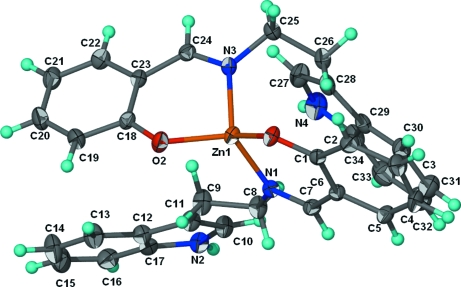
Thermal ellipsoid plot of Zn(C17H14ClN2O). Displacement ellipsoids are drawn at the 50% probability level, and H atoms are shown as spheres of arbitrary radii.

## supplementary crystallographic information

### Comment

We have recently reported the low-temperature structure of the zinc derivative of the ligand without any substituent, [C_17_H_15_N_2_O)_2_Zn] (Ng, 2008); the low-temperature structure is identical to the room-temperature structure (Chen *et al.*, 2007). The present compound has a chlorine substituent but this does not lead to significant changes to the bond dimensions of the central metal.

### Experimental

The Schiff base ligand was synthesized by the reaction of tryptamine (0.32 g, 2 mmol), 5-chlorosalicylaldehyde (0.24 g, 2 mmol) and zinc acetate (0.19 g, 1 mmol) in ethanol. There organic reagents were first heated for an hour. Zinc acetate was then added followed by excess of triethylamine (1 ml). Crystals were obtained by recrystallization from dimethylformamide.

### Refinement

All H atoms were located in a difference Fourier map, and were refined with distance restraints of C–H 1.00 Å and N–H 0.88 Å; their temperature factors were freely refined.

### Crystal data

**Table:**

[Zn(C_17_H_14_ClN_2_O)_2_]	*F*(000) = 1360
*M**_r_* = 660.87	*D*_x_ = 1.497 Mg m^−^^3^
Monoclinic, *C*2/*c*	Mo *K*α radiation, λ = 0.71073 Å
Hall symbol: -C 2yc	Cell parameters from 9956 reflections
*a* = 25.8989 (3) Å	θ = 2.5–31.2°
*b* = 5.4960 (1) Å	µ = 1.06 mm^−^^1^
*c* = 20.6138 (3) Å	*T* = 128 K
β = 91.801 (1)°	Block, colorless
*V* = 2932.73 (8) Å^3^	0.50 × 0.30 × 0.17 mm
*Z* = 4	

### Data collection

**Table:**

Bruker APEXII diffractometer	3352 independent reflections
Radiation source: medium-focus sealed tube	3023 reflections with *I* > 2σ(*I*)
graphite	*R*_int_ = 0.025
φ and ω scans	θ_max_ = 27.5°, θ_min_ = 1.6°
Absorption correction: multi-scan (*SADABS*; Sheldrick, 1996)	*h* = −33→33
*T*_min_ = 0.714, *T*_max_ = 0.840	*k* = −6→7
17664 measured reflections	*l* = −26→26

### Refinement

**Table:**

Refinement on *F*^2^	Primary atom site location: structure-invariant direct methods
Least-squares matrix: full	Secondary atom site location: difference Fourier map
*R*[*F*^2^ > 2σ(*F*^2^)] = 0.025	Hydrogen site location: inferred from neighbouring sites
*wR*(*F*^2^) = 0.100	All H-atom parameters refined
*S* = 1.21	*w* = 1/[σ^2^(*F*_o_^2^) + (0.0632*P*)^2^ + 1.1156*P*] where *P* = (*F*_o_^2^ + 2*F*_c_^2^)/3
3352 reflections	(Δ/σ)_max_ = 0.001
251 parameters	Δρ_max_ = 0.55 e Å^−^^3^
14 restraints	Δρ_min_ = −0.56 e Å^−^^3^

### Fractional atomic coordinates and isotropic or equivalent isotropic displacement parameters (Å^2^)

**Table:**

	*x*	*y*	*z*	*U*_iso_*/*U*_eq_	
Zn1	0.5000	0.49480 (5)	0.7500	0.01823 (11)	
Cl1	0.39490 (2)	0.33657 (10)	0.42675 (2)	0.03469 (14)	
O1	0.46240 (5)	0.6771 (2)	0.68555 (6)	0.0256 (3)	
N1	0.53085 (5)	0.2579 (3)	0.68736 (6)	0.0167 (3)	
C1	0.45075 (6)	0.5975 (3)	0.62688 (7)	0.0175 (3)	
N2	0.71557 (6)	−0.2875 (3)	0.76815 (7)	0.0235 (3)	
C2	0.41428 (7)	0.7308 (3)	0.58836 (8)	0.0226 (3)	
C3	0.39787 (7)	0.6548 (3)	0.52766 (8)	0.0230 (3)	
C4	0.41765 (7)	0.4409 (4)	0.50234 (8)	0.0216 (3)	
C5	0.45433 (7)	0.3096 (3)	0.53648 (8)	0.0206 (3)	
C6	0.47188 (6)	0.3836 (3)	0.59882 (7)	0.0167 (3)	
C7	0.51092 (6)	0.2301 (3)	0.63013 (8)	0.0181 (3)	
C8	0.56967 (6)	0.0809 (3)	0.71064 (8)	0.0186 (3)	
C9	0.62386 (7)	0.1885 (4)	0.70984 (11)	0.0305 (4)	
C10	0.66211 (7)	0.0247 (3)	0.74448 (10)	0.0233 (4)	
C11	0.68435 (7)	−0.1805 (3)	0.72056 (9)	0.0245 (4)	
C12	0.68028 (6)	0.0477 (3)	0.81090 (9)	0.0209 (3)	
C13	0.67219 (7)	0.2182 (4)	0.86012 (10)	0.0296 (4)	
C15	0.69689 (9)	0.1852 (4)	0.91969 (10)	0.0359 (5)	
C16	0.72995 (9)	−0.0135 (4)	0.93160 (11)	0.0344 (5)	
C17	0.73904 (7)	−0.1845 (4)	0.88415 (9)	0.0279 (4)	
C18	0.71359 (6)	−0.1517 (3)	0.82397 (8)	0.0205 (3)	
H2N	0.7331 (9)	−0.421 (3)	0.7639 (13)	0.042 (7)*	
H2	0.3996 (9)	0.884 (3)	0.6056 (11)	0.036 (6)*	
H3	0.3712 (7)	0.753 (4)	0.5031 (10)	0.033 (6)*	
H5	0.4670 (8)	0.155 (3)	0.5170 (11)	0.036 (6)*	
H7	0.5240 (7)	0.097 (3)	0.6024 (8)	0.017 (5)*	
H81	0.5617 (8)	0.033 (3)	0.7554 (6)	0.018 (5)*	
H82	0.5676 (9)	−0.067 (3)	0.6831 (10)	0.028 (5)*	
H91	0.6237 (11)	0.351 (3)	0.7316 (12)	0.052 (8)*	
H92	0.6344 (10)	0.217 (5)	0.6640 (6)	0.048 (8)*	
H11	0.6818 (9)	−0.251 (4)	0.6764 (6)	0.034 (6)*	
H13	0.6501 (8)	0.365 (3)	0.8521 (11)	0.038 (6)*	
H15	0.6893 (9)	0.299 (4)	0.9553 (9)	0.037 (6)*	
H16	0.7477 (10)	−0.026 (5)	0.9748 (8)	0.045 (8)*	
H17	0.7634 (8)	−0.323 (3)	0.8917 (12)	0.041 (7)*	

### Atomic displacement parameters (Å^2^)

**Table:**

	*U*^11^	*U*^22^	*U*^33^	*U*^12^	*U*^13^	*U*^23^
Zn1	0.02395 (17)	0.01667 (17)	0.01391 (15)	0.000	−0.00209 (10)	0.000
Cl1	0.0418 (3)	0.0423 (3)	0.0191 (2)	0.0051 (2)	−0.01249 (18)	−0.00457 (18)
O1	0.0394 (7)	0.0195 (6)	0.0173 (6)	0.0091 (5)	−0.0061 (5)	−0.0035 (5)
N1	0.0163 (6)	0.0165 (7)	0.0174 (6)	0.0011 (5)	−0.0005 (5)	0.0026 (5)
C1	0.0215 (7)	0.0158 (8)	0.0153 (7)	0.0005 (6)	−0.0003 (6)	0.0006 (6)
N2	0.0211 (7)	0.0231 (8)	0.0262 (7)	0.0056 (6)	−0.0016 (6)	−0.0016 (6)
C2	0.0264 (9)	0.0193 (8)	0.0222 (8)	0.0065 (7)	0.0000 (6)	0.0018 (6)
C3	0.0227 (8)	0.0247 (9)	0.0212 (8)	0.0039 (7)	−0.0029 (6)	0.0072 (7)
C4	0.0236 (8)	0.0268 (8)	0.0142 (7)	−0.0009 (7)	−0.0026 (6)	−0.0005 (6)
C5	0.0231 (8)	0.0214 (8)	0.0172 (7)	0.0029 (6)	−0.0001 (6)	−0.0021 (6)
C6	0.0185 (7)	0.0168 (8)	0.0148 (7)	0.0013 (6)	−0.0005 (5)	0.0007 (6)
C7	0.0186 (7)	0.0173 (8)	0.0186 (7)	0.0030 (6)	0.0006 (6)	−0.0001 (6)
C8	0.0183 (7)	0.0173 (8)	0.0201 (7)	0.0020 (6)	−0.0025 (6)	0.0032 (6)
C9	0.0186 (8)	0.0281 (10)	0.0444 (11)	−0.0022 (7)	−0.0044 (7)	0.0164 (9)
C10	0.0154 (7)	0.0226 (9)	0.0319 (10)	−0.0029 (6)	−0.0015 (7)	0.0075 (7)
C11	0.0206 (8)	0.0281 (9)	0.0246 (8)	−0.0026 (7)	−0.0040 (6)	0.0022 (7)
C12	0.0152 (7)	0.0179 (8)	0.0298 (9)	−0.0013 (6)	0.0030 (6)	0.0036 (7)
C13	0.0264 (9)	0.0199 (9)	0.0431 (11)	−0.0029 (7)	0.0094 (8)	−0.0036 (8)
C15	0.0408 (11)	0.0315 (11)	0.0359 (10)	−0.0118 (9)	0.0098 (8)	−0.0126 (9)
C16	0.0399 (11)	0.0382 (12)	0.0249 (10)	−0.0122 (8)	−0.0022 (8)	0.0001 (8)
C17	0.0265 (9)	0.0293 (10)	0.0275 (9)	−0.0020 (7)	−0.0043 (7)	0.0047 (7)
C18	0.0179 (7)	0.0194 (8)	0.0241 (8)	−0.0010 (6)	0.0010 (6)	0.0018 (6)

### Geometric parameters (Å, °)

**Table:**

Zn1—O1	1.907 (1)	C7—H7	0.994 (9)
Zn1—O1^i^	1.907 (1)	C8—C9	1.523 (2)
Zn1—N1	2.016 (1)	C8—H81	0.989 (9)
Zn1—N1^i^	2.016 (1)	C8—H82	0.994 (10)
Cl1—C4	1.745 (2)	C9—C10	1.502 (2)
O1—C1	1.312 (2)	C9—H91	0.998 (10)
N1—C7	1.282 (2)	C9—H92	1.004 (10)
N1—C8	1.469 (2)	C10—C11	1.366 (3)
C1—C2	1.419 (2)	C10—C12	1.439 (3)
C1—C6	1.427 (2)	C11—H11	0.990 (10)
N2—C18	1.374 (2)	C12—C13	1.402 (3)
N2—C11	1.383 (2)	C12—C18	1.415 (2)
N2—H2N	0.870 (10)	C13—C15	1.379 (3)
C2—C3	1.374 (2)	C13—H13	0.998 (10)
C2—H2	0.994 (10)	C15—C16	1.405 (3)
C3—C4	1.391 (3)	C15—H15	0.990 (10)
C3—H3	1.001 (10)	C16—C17	1.382 (3)
C4—C5	1.370 (2)	C16—H16	0.992 (10)
C5—C6	1.410 (2)	C17—C18	1.398 (2)
C5—H5	0.999 (10)	C17—H17	0.996 (10)
C6—C7	1.453 (2)		
			
O1—Zn1—O1^i^	116.62 (8)	N1—C8—H81	108.7 (12)
O1—Zn1—N1	95.57 (5)	C9—C8—H81	109.5 (12)
O1—Zn1—N1^i^	125.55 (6)	N1—C8—H82	109.4 (14)
O1^i^—Zn1—N1	125.55 (6)	C9—C8—H82	110.3 (13)
O1^i^—Zn1—N1^i^	95.57 (5)	H81—C8—H82	107.8 (18)
N1—Zn1—N1^i^	99.56 (8)	C10—C9—C8	110.87 (15)
C1—O1—Zn1	124.38 (11)	C10—C9—H91	109.5 (17)
C7—N1—C8	118.25 (14)	C8—C9—H91	109.0 (16)
C7—N1—Zn1	120.68 (11)	C10—C9—H92	110.3 (16)
C8—N1—Zn1	119.96 (10)	C8—C9—H92	110.3 (15)
O1—C1—C2	118.25 (15)	H91—C9—H92	107 (2)
O1—C1—C6	124.63 (15)	C11—C10—C12	106.68 (16)
C2—C1—C6	117.12 (14)	C11—C10—C9	127.04 (19)
C18—N2—C11	109.06 (15)	C12—C10—C9	126.20 (17)
C18—N2—H2N	125.3 (18)	C10—C11—N2	109.81 (16)
C11—N2—H2N	125.7 (18)	C10—C11—H11	129.7 (14)
C3—C2—C1	122.28 (16)	N2—C11—H11	120.4 (14)
C3—C2—H2	118.2 (14)	C13—C12—C18	118.94 (17)
C1—C2—H2	119.6 (14)	C13—C12—C10	134.06 (17)
C2—C3—C4	119.47 (15)	C18—C12—C10	106.99 (15)
C2—C3—H3	119.0 (14)	C15—C13—C12	118.76 (19)
C4—C3—H3	121.5 (14)	C15—C13—H13	120.0 (14)
C5—C4—C3	120.68 (16)	C12—C13—H13	121.2 (14)
C5—C4—Cl1	119.65 (14)	C13—C15—C16	121.40 (19)
C3—C4—Cl1	119.66 (13)	C13—C15—H15	118.7 (15)
C4—C5—C6	120.98 (16)	C16—C15—H15	119.8 (15)
C4—C5—H5	118.2 (14)	C17—C16—C15	121.4 (2)
C6—C5—H5	120.8 (14)	C17—C16—H16	120.4 (16)
C5—C6—C1	119.38 (14)	C15—C16—H16	118.2 (16)
C5—C6—C7	115.93 (14)	C16—C17—C18	117.02 (18)
C1—C6—C7	124.69 (14)	C16—C17—H17	121.9 (15)
N1—C7—C6	126.44 (15)	C18—C17—H17	121.1 (15)
N1—C7—H7	118.8 (12)	N2—C18—C17	130.10 (17)
C6—C7—H7	114.6 (12)	N2—C18—C12	107.45 (15)
N1—C8—C9	111.15 (14)	C17—C18—C12	122.43 (17)
			
O1^i^—Zn1—O1—C1	153.77 (15)	C1—C6—C7—N1	2.3 (3)
N1—Zn1—O1—C1	19.16 (14)	C7—N1—C8—C9	104.80 (18)
N1^i^—Zn1—O1—C1	−86.78 (15)	Zn1—N1—C8—C9	−87.15 (16)
O1—Zn1—N1—C7	−18.84 (14)	N1—C8—C9—C10	169.91 (16)
O1^i^—Zn1—N1—C7	−147.38 (12)	C8—C9—C10—C11	79.8 (2)
N1^i^—Zn1—N1—C7	108.67 (14)	C8—C9—C10—C12	−96.6 (2)
O1—Zn1—N1—C8	173.41 (12)	C12—C10—C11—N2	−0.1 (2)
O1^i^—Zn1—N1—C8	44.87 (14)	C9—C10—C11—N2	−177.12 (16)
N1^i^—Zn1—N1—C8	−59.09 (10)	C18—N2—C11—C10	0.3 (2)
Zn1—O1—C1—C2	167.80 (12)	C11—C10—C12—C13	178.99 (19)
Zn1—O1—C1—C6	−11.6 (2)	C9—C10—C12—C13	−4.0 (3)
O1—C1—C2—C3	−176.76 (17)	C11—C10—C12—C18	−0.05 (19)
C6—C1—C2—C3	2.7 (3)	C9—C10—C12—C18	176.96 (16)
C1—C2—C3—C4	−0.4 (3)	C18—C12—C13—C15	−0.2 (3)
C2—C3—C4—C5	−1.9 (3)	C10—C12—C13—C15	−179.14 (19)
C2—C3—C4—Cl1	177.25 (14)	C12—C13—C15—C16	0.4 (3)
C3—C4—C5—C6	1.8 (3)	C13—C15—C16—C17	−0.1 (3)
Cl1—C4—C5—C6	−177.31 (13)	C15—C16—C17—C18	−0.4 (3)
C4—C5—C6—C1	0.5 (3)	C11—N2—C18—C17	−178.80 (18)
C4—C5—C6—C7	−179.84 (16)	C11—N2—C18—C12	−0.31 (19)
O1—C1—C6—C5	176.72 (16)	C16—C17—C18—N2	178.95 (18)
C2—C1—C6—C5	−2.7 (2)	C16—C17—C18—C12	0.7 (3)
O1—C1—C6—C7	−2.9 (3)	C13—C12—C18—N2	−178.99 (16)
C2—C1—C6—C7	177.69 (15)	C10—C12—C18—N2	0.22 (19)
C8—N1—C7—C6	179.43 (15)	C13—C12—C18—C17	−0.4 (3)
Zn1—N1—C7—C6	11.5 (2)	C10—C12—C18—C17	178.85 (16)
C5—C6—C7—N1	−177.31 (16)		

Symmetry codes: (i) −*x*+1, *y*, −*z*+3/2.
